# Manic-Like Psychosis Associated with Elevated Trough Tacrolimus Blood Concentrations 17 Years after Kidney Transplant

**DOI:** 10.1155/2013/926395

**Published:** 2013-05-16

**Authors:** Giuseppe Bersani, Pietropaolo Marino, Giuseppe Valeriani, Valentina Cuoco, Claudia Zitelli, Claudia Melcore, Francesco Saverio Bersani

**Affiliations:** ^1^Department of Medical-Surgical Sciences and Biotechnologies, UOD Fiorini Hospital, Sapienza University of Rome, Viale dell'Universitá No. 30, 00185 Rome, Italy; ^2^Department of Neurology and Psychiatry, Sapienza University of Rome, Viale dell'Universitá No. 30, 00185 Rome, Italy

## Abstract

Several neurological side effects induced by tacrolimus are described in the scientific literature, ranging from mild neurological symptoms to delirium and psychosis. We report the case of a 46-year-old man with no prior psychiatric history who suddenly manifested manic-like psychosis associated with elevated trough tacrolimus blood concentrations 17 years after kidney transplant. The use of antipsychotics may improve the severity of symptoms; but in order to obtain a complete remission, the reduction in the dose of tacrolimus, or its replacement with alternative immunosuppressant therapies, is recommended.

## 1. Introduction

Calcineurin-inhibitor Tacrolimus (FK506; Fujisawa, Deerfield, IL, USA) is considered one of the mainstays of posttransplant immunosuppression [1]. Discovered in 1984, it is a macrolide lactone extracted from *Streptomyces tsukubaensis* as an alternative to cyclosporine [2].

Tacrolimus mechanism of action is fulfilled through the binding with the cytoplasmic protein macrophilin 12 and the consequent inhibition of calcium-dependent phosphatase calcineurin, which is followed by the blockade of the transcription factor NF-AT [3].

Tacrolimus has a narrow therapeutic window with wide interindividual variability in pharmacokinetics and clearance [4, 5]. Its availability mostly depends on the activity of hepatic and intestinal CYP3A4, and its active transport is mediated by intestinal P-glycoprotein [6]. Less than 1% of the drug is excreted unchanged in the urine [7]. Based on FK506 consensus reports by Jusko et al. [8] and Wong [9], its therapeutic ranges in kidney transplanted patients should be 10–15 *μ*g/L in the first 6 months of treatment; 8–12 *μ*g/L in the following semester; and 5–10 *μ*g/L as maintenance therapy after 1 year. 

Even if this highly beneficial drug is critical for post-transplant survival, a significant number of transplant recipients experience neurological side effects, with potential severe impact on mental status and cognition. 

Numerous cases of mild neurological side effects including tremors, paresthesias, and headache [10] have been described, while more severe neurological and psychiatric side effects seem to occur more rarely (Table 1).

Later, we describe the case of a 46-year-old man who developed a manic-like psychosis as a consequence of high blood concentrations of tacrolimus.

## 2. Case Report

Mr. MP was a 46-year-old man who underwent left kidney transplant in 1996; therefore, he was treated with the following immunosuppressive therapy: prednisone 5 mg/day, mycophenolate mofetil 500 mg/day, and tacrolimus (since 2000 on the dose of 6.5 mg/day). He never had any psychiatric or neurological disease before 2012.

At the beginning of September 2012, he experienced nonspecific symptoms including asthenia, diarrhea, subjective vertigo, and mild dysarthria. Electroencephalography (EEG) highlighted slight non-specific abnormalities in central regions bilaterally; a cerebral magnetic resonance imaging (MRI) did not identify any focal brain or cerebellar lesions or alterations of ventricular system. 

On September 29, Mr. MP suddenly developed intense psychomotor agitation and delusional ideas polarized on mystical issues. The patient was lucid and well oriented to time, person, and place. He perceived “hints” provided by a spiritual entity (Saint Pio of Pietrelcina) about lottery numbers and football results. Driven by this delusion, Mr. MP convinced his wife to see the drawing of the lottery numbers and to watch several football games; later, he spent several hours in front of the television waiting for new lotteries and new matches, forcing his wife to be with him and to be “ready.” 

This psychopathological condition lasted several days: the patient presented the same mystical delusions, euphoric mood, psychomotor agitation, almost total insomnia, increased energy, and increased productivity of speech (despite the mild dysarthria). In addition, gastrointestinal and neurological symptoms persisted, also with some falls on the floor, vertigo related.

Mr. MP underwent the first psychiatric evaluation on October 5, at the emergency room of Fiorini University Hospital of Sapienza University of Rome. The severity of the psychopathological condition and the absence of laboratory and instrumental data able to explain the psychiatric symptomatology, led to the initiation of a pharmacological therapy with valproic acid up to 600 mg/day and olanzapine up to 7.5 mg/day.

The patient was then reevaluated every week during the next two weeks at the psychiatric outpatient clinic of the Hospital; he showed a gradual reduction of agitation and insomnia, but delusional ideas did not improve.

On October 22 Mr. MP was hospitalized for the onset of tonic-clonic seizures at the neurological ward of the same Hospital. The EEG examination confirmed a slight alteration of brain electrical activity in frontotemporal bilateral regions; cerebral MRI again excluded pathological focal lesions; and cerebrospinal fluid examination was normal.

At the admission, serum creatinine level was 270.5 *μ*mol/L and glomerular filtration rate (GFR) was 22.13 mL/min/1.73 m^2^. Although renal function was bad, it did not seem to justify the psychopathological symptomatology; moreover, renal function was similar to previous evaluations: in the previous years the serum creatinine level ranged between 212 and 248 *μ*mol/L and GFR ranged between 28 and 24.20 mL/min/1.73 m^2^. 

In the absence of other evident metabolic abnormalities, the medical team explored the possibility of an iatrogenic condition. The patient presented clinical documentation of the monitoring of tacrolimus blood concentrations. The last measurement was carried out on July 31, 2012, before the onset of clinical manifestations; at that moment, tacrolimus concentration was 4.5 ng/mL. From the beginning of the treatment with tacrolimus in 2000, the blood concentration of the drug ranged between 4 and 8 ng/mL. The measurement of tacrolimus concentration performed during the hospitalization, on October 25, showed a concentration of 13.8 ng/mL; the patient did not remember to have never reached such an high concentration in the past years.

After the laboratory results, the decision to reduce the dosage of tacrolimus to 3 mg/day was taken; as a consequence, the blood concentration of the drug decreased to 2.9 ng/mL in a few days.

Delusional ideas gradually disappeared; a progressive remission of diarrhea and neurological symptoms was simultaneously recorded. 

At the discharge, on November 16, tacrolimus therapy was restored at 4 mg/day; the treatment with valproic acid and olanzapine was gradually suspended with no subsequent psychopathological relapses.

## 3. Discussion

Although late-onset psychotic episodes are described in the literature [11], the patient's medical history (nephropathy and immunosuppressive therapy) and the combination of neurological signs and symptoms (dysarthria, subjective vertigo, and seizures) guide our diagnosis to a psychotic disorder due to general medical condition rather than an endogenous psychosis. Physicians' first hypothesis for the etiology of such symptoms was represented by neurological diseases, ranging from vascular to neoplastic or infectious causes, but laboratory and instrumental data showed no pathological findings that could justify the clinical manifestations presented. On the other hand, the medical condition of renal failure is related rather to pictures of Delirium, that is, an altered state of consciousness that influence the awareness of the external environment. However, in the case described the state of consciousness is preserved, and the patient was perfectly oriented in the three dimensions (time, space, and person). 

The hypothesis of a iatrogenic origin of the symptoms of Mr. MP, initially not considered by physicians as the immunosuppressive treatment had remained unchanged for many years (both in the selection of molecules and in the choice of dosage), may justify both the neurological and psychiatric manifestations. 

In the course of tacrolimus treatment, it is not uncommon to observe oscillations of its blood concentration; the most frequent causes may be drug interactions (e.g., with macrolide antibiotics, azole antifungals, or calcium channel blockers) [12]; reduced liver function or changes in gastrointestinal transit time (e.g., diarrhea, as reported by our patient in the period preceding the psychopathological onset, and persistent also during the hospitalization) for reduced both hepatic and intestinal CYP3A4 and P-glycoprotein activities, determinants of tacrolimus bioavailability [13].

Only limited reports on psychiatric symptoms induced by tacrolimus are present in the medical literature. Neurotoxicity and delirium have been reported to occur during tacrolimus treatment in hepatic and kidney allograph [14]; hallucinations, anxiety, paranoid delusions, and dissociative fugues [15, 16] have also been associated with elevated tacrolimus blood concentrations.

The differences between the present case and other cases of neuropsychiatric side effects of tacrolimus are mainly two. Firstly, Mr. MP showed no disorientation, no disturbance of consciousness, nor cognitive deficits; delirium and confusion were thus completely absent. Secondly, Mr. MP, while not satisfying the clinical criteria for bipolar or schizoaffective disorder, presented delusions associated with behavioural and affective symptoms similar to those of mania states: euphoric mood, psychomotor agitation, insomnia, increased energy, accelerated mental activity, and pressured speech; to our knowledge, this is the first reported case of manic-like psychosis associated with toxicity through tacrolimus blood concentrations. 

Although manic-like psychosis may be a rare adverse effect, it can have significant impact on the long-term prognosis and treatment in transplant recipients. The appropriate management of these clinical pictures cannot be separated from the evaluation of blood concentrations of tacrolimus; psychiatric treatment in part may provide an improvement in psychopathology, but its resolution has to be linked to a review of immunosuppressive therapy; some authors, in order to reduce the neurotoxicity, recommend that tacrolimus levels are kept below 10 ng/mL after the second transplant month in straightforward transplants [10]; a possible alternative is the replacement of tacrolimus with cyclosporine [17].

## Figures and Tables

**Table 1:** Neurologic complications of tacrolimus therapy in transplants patients (1, 2, 3).

Common side effects (up to 60%)	Mild neurotoxic effects (headache, paresthesias, tremor, sleep disturbances, photophobia, and dysesthesias)

Uncommon side effects (5–8%)	Severe neurologic symptoms (confusion, seizures, posterior reversible encephalopathy, akinetic mutism, and dysarthria)

Rare side effects (0.4–5.2%)	Psychosis, coma, aphasia, and intracranial haemorrhage
